# Community ART Support Groups in Mozambique: The Potential of Patients as Partners in Care

**DOI:** 10.1371/journal.pone.0166444

**Published:** 2016-12-01

**Authors:** Kebba Jobarteh, Ray W. Shiraishi, Inacio Malimane, Paula Samo Gudo, Tom Decroo, Andrew F. Auld, Vania Macome, Aleny Couto

**Affiliations:** 1 Division of Global HIV/AIDS, Center for Global Health, Centers for Disease Control and Prevention, Maputo, Mozambique; 2 Division of Global HIV/AIDS, Center for Global Health, Centers for Disease Control and Prevention, Atlanta, United States of America; 3 Medical Department, Médecins Sans Frontières, Operational Centre Brussels, Brussels, Belgium; 4 Mozambique Ministry of Health, Maputo, Mozambique; San Antonio Military Medical Center, UNITED STATES

## Abstract

**Background:**

High rates of attrition are stymying Mozambique’s national HIV Program’s efforts to achieve 80% treatment coverage. In response, Mozambique implemented a national pilot of Community Adherence and Support Groups (CASG). CASG is a model in which antiretroviral therapy (ART) patients form groups of up to six patients. On a rotating basis one CASG group member collects ART medications at the health facility for all group members, and distributes those medications to the other members in the community. Patients also visit their health facility bi-annually to receive clinical services.

**Methods:**

A matched retrospective cohort study was implemented using routinely collected patient-level data in 68 health facilities with electronic data systems and CASG programs. A total of 129,938 adult ART patients were registered in those facilities. Of the 129,938 patients on ART, 6,760 were CASG members. A propensity score matched analysis was performed to assess differences in mortality and loss to follow-up (LTFU) between matched CASG and non-CASG members. Propensity scores were estimated using a random-effects logistic regression model. The following covariates where included in the model: sex, educational status, WHO stage, year of ART initiation, age, CASG eligibility, CD4 cell count category, weight, and employment status.

**Results:**

Non-CASG participants had higher LTFU rates (HR 2.356; p = 0.04) than matched CASG participants; however, there were no significant mortality differences between CASG and non-CASG participants. Compared with the full cohort of non-CASG members, CASG members were more likely to be female (74% vs. 68%), tended to have a lower median CD4 counts at ART initiation (183 cells/m3 vs. 200cells/m3) and be less likely to have a secondary school education (15% vs. 23%).

**Conclusion:**

ART patients enrolled in CASG were significantly less likely to be LTFU compared to matched patients who did not join CASG. CASG appears to be an effective strategy to decrease LTFU in Mozambique’s national ART program.

## Introduction

Mozambique ranks 178 of 187 on the 2013 United Nations Development Program Human Development Index. [1] With an adult HIV prevalence of 11·5% and an estimated 1·6 million people living with HIV, Mozambique has the fifth largest HIV epidemic in sub-Saharan Africa (SSA).[2] With 3 doctors and 21 nurses per 100,000 inhabitants, Mozambique also has one of the worst global shortages of human resources for health.[3,4] Despite the mismatch between the scale of the epidemic and the resources available to combat it, Mozambique has succeeded in realizing a massive scale-up of the national antiretroviral therapy (ART) program.

In the past few years, the number of people on ART in Mozambique has more than doubled, from 270,000 patients on ART in 2011 to 646,000 people living with HIV (PLHIV) on ART in December 2014.[5] However, low retention rates, currently at 67% in the national 12-month cohort, threaten to undo the remarkable gains in ART enrolment that Mozambique has achieved in the past few years.[6] Most of the attrition—87% according to a recent evaluation—is due to loss to follow-up (LTFU), and 13% are reported dead. Furthermore, an estimated 20–60% of patients LTFU are presumed dead, likely due to medication non-adherence and consequent rapid progression of disease. [7,8]

In an effort to tackle this problem and achieve the government’s goal of treating almost 1 million HIV-infected people by 2017, the Ministry of Health (MOH) adapted a community based ART model called Community Adherence and Support Groups (CASG). This model was piloted in the central province of Tête where after a reported median follow-up time of 13 months, retention among CASG members was 97.5% in CASG, 0.2% were LTFU and 2.3% had died.[9] Guidelines were subsequently developed by the MoH and their partners to scale-up CASG as a national pilot.[10] This model takes the structural constraints of patients and the health system into account and empowers patients to partner with one another and the health system to improve their own care. The CASG model groups patients together in order to establish a rotational drug collection and distribution system in the community, with one patient collecting ART for up to five other patients on a monthly basis and distributing those antiretrovirals to them in the community. Participation in CASG reduces time spent travelling and queuing for monthly ART refills. In addition patients meet in their communities, share treatment experiences, and support each other.[11]

The Tête study did not compare outcomes of patients in CASG with outcomes of patients in conventional care, thus it is currently unknown whether these patients differ in terms of LTFU and mortality. The current study will evaluate the impact of this large-scale CASG pilot by matching patients in CASG with patients in conventional care and comparing LTFU and mortality between the two groups.

## Methods

### CASG dynamic

CASGs are self-forming groups, designed to support up to six HIV-infected adults on ART. To be eligible, patients must be stable on ART for > 6 months, have a CD4 count > 200 cells/μL, not have active World Health Organization (WHO) stage III or IV conditions and not be pregnant. Once a CASG is formed, health facility staff engages group members in a learning session about the dynamic of the group. Monthly, CASG members delegate a representative to pick up ART at the health facility and on a rotating basis, each CASG member travels to the health facility every six months (in a six-member group). The day before a clinic visit, members meet to discuss the previous month’s challenges and conduct pill counts. Group members also complete a screening questionnaire designed to relay clinical information to the clinician at the health facility about all group members. On the visit day, the CASG representative has blood for a CD4 count drawn, has a clinical consultation, shares the screening questionnaire with the clinician and collects medicines for the group. That evening the representative reconvenes with the group in the community to distribute ARVs and discuss information provided by the health facility staff. Any member whose screening is of concern to the clinician is instructed to return immediately to the health facility for a clinical consultation.

### CASG national roll out

Beginning in 2011 The MoH transformed the CASG from a small-scale intervention in central Mozambique into a national pilot being implemented in 69 health facilities in all 11 provinces in Mozambique. The clinics represent facilities that are a mix of urban and rural, and high and low volume ART clinics. Based on the results of this pilot, reported here, the MoH expanded the CASG program from a pilot to a national strategy. At the time of writing there were over 37,000 patients enrolled in CASGs and the dynamic will be available in all 844 health facilities offering ART in Mozambique.

### Study Design and Population

This is a matched retrospective cohort study. Data was collected from facilities with Electronic Patient Tracking Systems (EPTS). By April 2014, 170 (60%) of 288 adult ART facilities in 7 of Mozambique’s 11 Provinces were using EPTS and of those, 68 sites were offering the CASG program to their patients. All covariates analyzed in this evaluation came from the EPTS databases. The EPTS data is an electronic version of the national HIV program clinical patient forms. All of the information from the clinical patient forms is back entered by PEPFAR supported staff into the EPTS databases. CASG relevant information is part of the routinely collected information in government facilities and the EPTS reflects that data as well. At the time of analysis, PEPFAR partners supported great than 90% of Mozambique’s ART facilities.

For this analysis, adults ≥15 years old at ART initiation, who started ART during 2004–2014 at facilities offering CASG services were eligible. The CASG cohort included patients who enrolled in CASG services after December 31, 2010. Facility-level databases were closed at the time of the most recent data transfer from the facility prior to starting analysis in April 2014.

Data from CASG and non-CASG patients were analyzed to assess their CASG eligibility based on age (≥15), CD4 cell count (>200 cells/μL) and time on ART (>6 months). WHO stage and pregnancy status were not included in the eligibility assessment, as these variables are not routinely updated in EPTS. Age and time on ART were calculated at each CD4 cell count, and patients were considered CASG-eligible if they met the eligibility criteria at any point during ART treatment.

In real terms, criteria for entry into CASG groups were not always strictly adhered to by the staff implementing this national CASG pilot. As such, some CASG groups contained a small number of patients who were not eligible for CASG enrolment. Since the purpose of this evaluation is to analyze the impact of CASG in a real world programmatic setting, these patients were included in the overall description of the CASG cohort. To determine the impact of inclusion of ineligible patients in the CASG dynamic, we separately compared their outcomes with eligible CASG members.

The primary ART outcomes of interest were mortality and LTFU. Patients were considered LTFU if ≥60 days late for their next scheduled medication pick-up appointment. Mortality ascertainment occurred largely through passive reporting.

### Analytic Methods

A propensity score matched analysis was performed to assess differences in mortality and LTFU between matched CASG and non-CASG members. Propensity scores were estimated using random-effects logistic regression, with random-effects specified on the intercept for reporting facility. Covariates in the logistic regression model were selected a priori based on their relationship to CASG eligibility and membership, and included sex, educational status, WHO stage, year of ART initiation, age group, CASG eligibility, CD4 cell count category, weight (kg) and employment status. CASG eligibility was a matching criterion because some CASG ineligible patients were found to have joined a CASG. Missing covariate data were imputed using multiple imputation by fully conditional specification (FCS).[12] Ten imputed datasets were constructed using the mi impute procedure in STATA (StataCorp. 2013. *Stata 13 Base Reference Manual*. College Stata, TX: Stata Press). The imputation model included CASG membership, ART reporting facility and the above covariates (i.e., sex, educational status [missingness = 20%], WHO stage [missingness = 19%], year of ART initiation, age group [missingness = 0.2%], CASG eligibility, CD4 cell count category [missingness = 31%], weight (kg) [missingness = 29%], and employment status [missingness = 16%]). Missing data were assumed *missing at random* (MAR).[13] The results from the first iteration of the augmented across method are presented in the text of the manuscript (see below).

Estimated propensity scores were averaged within individuals, and a modified version of the psmatch2 package was used to perform without replacement 1:1 nearest-neighbor caliper matching, the width of the caliper was set at 0.25 times the standard deviation of the estimated propensity score.[14] Matches were required to be from the same reporting facility as the CASG participant. The psmatch2 package was modified to replace matches who had outcome dates prior to the CASG participant’s enrollment in CASG with someone who had an outcome date greater than or equal to the CASG participant’s enrollment in CASG. The program would iterate a maximum of 10 times until either a suitable match was identified or no match was found.

To assess covariate balance between CASG and non-CASG participants, we calculated standardized differences for each covariate included in the logistic regression model using the mpbalchk package.[15, 16] The standardized differences for the propensity score matched cohort were compared to the full cohort. Covariate balance was assessed for each iteration of the augmented across method (see below); however, for ease of interpretation, we summarized the results from the 100 imputed datasets. A standardized difference greater than 0.10 or 10% was considered to represent a meaningful covariate imbalance between the CASG and non-CASG groups.[17]

A stratified Cox regression analysis using a clustered sandwich variance estimator was used to assess differences in LTFU and mortality between CASG and non-CASG participants and account for matching and clustering within facilities.[18] The origin was specified as the CASG start date for CASG participants; non-CASG participants were assigned the CASG start date of their match. For CASG members who joined CASG prior to starting ART, the origin was specified as their ART start date. CASG members who left a group were analyzed according to their original group membership, similar to an intention-to-treat (ITT) analysis for randomized control trials.

We used the augmented across method for its potential to reduce bias by repeating the above steps 10 times and then summarizing the resultant 10 treatment effects using Rubin’s rules. [19,20]

A competing risks model was used to estimate yearly mortality and LTFU between CASG and matched non-CASG participants. Estimates at discrete time points were averaged across imputations.

A Cox proportional hazards model using a clustered sandwich variance estimator was used to assess differences in LTFU and mortality between eligible and ineligible CASG participants.

Data were analyzed using STATA 13 (StataCorp, 2009, Stata Statistical Software, Release 13, College Station, TX: StataCorp LP).

### Ethics Approval

This study was approved by the Mozambican National Scientific Ethics Committee and the CDC Institutional Review Board. All patient level data was de-identified and anonymized prior to analysis. As a retrospective analysis, prior written informed consent was not obtained.

## Results

**Fig 1:**
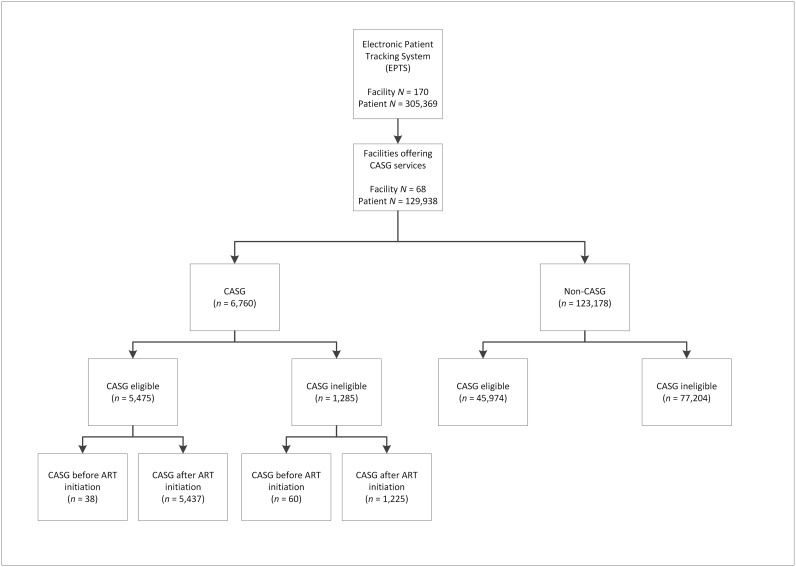
Study flow diagram.

As of April 2014, EPTS contained patient-level data on 305,369 ART patients from 170 ART treatment sites in Mozambique (Fig 1). Of the 170 facilities with EPTS, 68 had active CASG programs at the time of database closure. Of the 129,938 patients on ART at these facilities, 6,760 (5.2%) were members of CASG with two or more members on ART.

### CASG Eligibility

Of the 6,760 CASG participants, 81% (5,475) were CASG eligible, while of the 123,178 non-CASG participants, 37% (45,974) were eligible. Ninety-eight CASG participants joined CASG prior to ART initiation.

Since this is a retrospective program evaluation, there were 1,285 ART patients enrolled in CASGs who did not, at any time during the period of analysis, meet eligibility criteria. This inclusion is a result of non-adherence to eligibility criteria by program staff, but given the nature of this evaluation, the authors felt it was important to include this population in the analysis, since it reflects the operational reality of rolling out a novel intervention. In order to determine the impact of this inclusion and determine whether there were differences in the outcomes of these patients, a population-specific sub-analysis was conducted and the results reported in the following section.

### Predictors of CASG membership

Among the 129,938 patients at 68 facilities providing CASG services, females were significantly more likely to join CASG than males (adjusted odds ratio [aOR]: 1.4; 95% confidence interval [CI]: 1.3–1.5; Table 1). Patients with secondary education were significantly less likely to join CASG (aOR: 0.8; 95% CI: 0.7–0.9) compared with patients with no formal education. Patients with university education were marginally less likely to join CASG compared with patients with no formal education (aOR: 0.6; 95% CI: 0.4–1.1). Compared with patients with WHO Stage I disease at ART initiation, patients with WHO Stage IV disease were marginally less likely to join CASG (aOR: 0.9; 95% CI: 0.8–1.0). Compared with the youngest age group (15–19), patients in older age groups had significantly higher odds of joining CASG. Patients with CD4 cells counts of 200–499 cells/uL at ART initiation were significantly more likely to join CASG compared with patients with CD4 cell counts <50 cells/uL at the time of ART initiation. Patients who were eligible for CASG based on age, CD4 cell count, and time on ART had significantly higher odds of joining CASG (aOR: 2.6; 95% CI: 2.4–2.8). Compared with unemployed patients, employed patients were less likely join CASG (aOR: 0.9; 95% CI: 0.8–1.0).

**Table 1:** Predictors of CASG membership (N = 129,938 at 68 facilities providing CASG Services).

	aOR (95% CI)	p-value
**Year of ART Initiation**		
2004/2005	ref.	--
2006	0.697 (0.43, 1.128)	0.141
2007	0.605 (0.378, 0.967)	0.036
2008	0.581 (0.364, 0.928)	0.023
2009	0.575 (0.361, 0.918)	0.02
2010	0.510 (0.32, 0.814)	0.005
2011	0.338 (0.212, 0.54)	<0.001
2012	0.150 (0.094, 0.24)	<0.001
2013/2014	0.028 (0.017, 0.046)	<0.001
**Sex**		
Male	ref.	--
Female	1.403 (1.308, 1.505)	<0.001
**Education**		
None	ref.	
Other	0.877 (0.601, 1.278)	0.494
Primary	0.987 (0.905, 1.076)	0.764
Secondary	0.820 (0.726, 0.926)	0.002
University	0.634 (0.374, 1.077)	0.091
**WHO Stage**		
I	ref.	--
II	1.004 (0.927, 1.087)	0.927
III	0.960 (0.879, 1.049)	0.361
IV	0.888 (0.776, 1.016)	0.082
**Age group**		
15–19	ref.	--
20–24	1.394 (1.099, 1.77)	0.006
25–29	1.824 (1.447, 2.299)	<0.001
30–34	2.113 (1.676, 2.663)	<0.001
35–39	2.465 (1.951, 3.114)	<0.001
40–44	2.824 (2.231, 3.576)	<0.001
45–49	2.704 (2.124, 3.441)	<0.001
50–54	2.816 (2.199, 3.606)	<0.001
55–59	2.314 (1.757, 3.046)	<0.001
60–64	2.466 (1.827, 3.329)	<0.001
65+	2.341 (1.642, 3.337)	<0.001
**CD4 count category**		
<50 cells/uL	ref.	--
50–199 cells/uL	1.092 (0.974, 1.224)	0.131
200–349 cells/uL	1.235 (1.091, 1.397)	0.001
350–499 cells/uL	1.181 (1.007, 1.386)	0.041
> = 500 cells/uL	1.172 (0.94, 1.461)	0.154
**Weight (baseline)**	1.002 (0.998, 1.005)	0.352
**CASG eligible**		
No	ref.	--
Yes	2.580 (2.393, 2.782)	<0.001
**Employment status**		
Unemployed	ref.	--
Student	1.015 (0.818, 1.258)	0.894
Work at home/housewife	0.989 (0.864, 1.133)	0.878
Farmer	1.103 (0.957, 1.27)	0.176
Employed	0.865 (0.753, 0.994)	0.041

Note. Results from first 10 imputations.

### Propensity Score Matched Cohort

Compared with non-CASG participants in the full cohort, a larger percentage of CASG patients were female (68% vs. 74%), lacked formal education (14% vs. 22%), had WHO Stage III disease at ART initiation (30% vs. 39%), were CASG eligible (39% vs. 80%) and were farmers (17% vs. 25%; Table 2). Compared with non-CASG participants, CASG patients were less likely to have completed secondary education (23% vs. 15%), have WHO stage I disease at ART initiation (40% vs. 28%) and be employed (28% vs. 23%). CASG patients also tended to be older, have lower CD4 cell counts at ART initiation, and belong to older ART cohorts. There were no appreciable differences between the CASG and non-CASG propensity score matched cohort.

**Table 2:** Standardized difference between CASG and non-CASG participants in the full and propensity score matched cohort.

	Full Cohort			Propensity Score Matched Cohort		
	CASG			CASG		
	Yes (N = 6,760)	No (N = 123,178)	Standardized Difference	Yes (N = 6,648*)	No (N = 6,648*)	Standardized Difference
**Year of ART Initiation**						
2004/2005	0%	0%	0.031	0%	0%	-0.001
2006	3%	1%	0.135	3%	3%	-0.029
2007	12%	4%	0.281	12%	10%	0.039
2008	14%	5%	0.315	14%	13%	0.025
2009	18%	7%	0.316	18%	18%	0.003
2010	21%	10%	0.287	20%	21%	-0.002
2011	18%	15%	0.064	18%	18%	-0.010
2012	13%	27%	-0.356	13%	15%	-0.040
2013/2014	2%	30%	-0.814	2%	2%	0.012
**Sex**						
Male	26%	32%	-0.147	26%	26%	-0.003
Female	74%	68%	0.147	74%	74%	0.003
**Education**						
None	22%	14%	0.220	22%	21%	0.017
Other	1%	1%	-0.030	1%	0%	0.005
Primary	62%	62%	0.013	62%	63%	-0.012
Secondary	15%	23%	-0.206	15%	15%	0.002
University	0%	1%	-0.087	0%	1%	-0.027
**WHO Stage**						
I	28%	40%	-0.259	28%	28%	-0.006
II	28%	25%	0.061	27%	28%	-0.011
III	38%	30%	0.178	39%	38%	0.004
IV	6%	5%	0.049	6%	5%	0.026
**Age** (Mean)	37.68	35.09	0.248	37.64	37.47	0.016
**Age Group**						
15–19	1%	3%	-0.131	1%	1%	-0.029
20–24	7%	12%	-0.152	7%	7%	0.007
25–29	16%	20%	-0.108	16%	15%	0.019
30–34	18%	20%	-0.055	18%	19%	-0.018
35–39	17%	15%	0.040	16%	17%	-0.015
40–44	16%	11%	0.141	15%	15%	0.006
45–49	11%	8%	0.112	11%	11%	-0.005
50–54	8%	6%	0.094	8%	8%	0.003
55–59	3%	3%	0.036	3%	3%	0.050
60–64	2%	2%	0.012	2%	2%	-0.020
65+	1%	1%	-0.009	1%	1%	0.003
**CD4 Count** (Mean)	202.29	228.70	-0.179	202.37	205.16	-0.023
**sqrt(CD4 Count)** (Mean)	13.54	14.15	-0.123	13.54	13.66	-0.028
**CD4 Count Category**						
< 50 cell/uL	8%	10%	-0.071	8%	7%	0.031
50–199 cells/uL	44%	37%	0.128	44%	44%	-0.003
200–349 cells/uL	41%	39%	0.047	41%	41%	0.005
350–499 cells/uL	4%	8%	-0.137	5%	6%	-0.047
≥ 500 cells/uL	3%	6%	-0.163	3%	2%	0.007
**Weight** (Mean)	56.51	57.62	-0.123	56.54	56.78	-0.027
**Weight Category**						
45–60	74%	70%	0.100	74%	74%	0.016
>60	26%	30%	-0.100	26%	26%	-0.016
**CASG Eligible**						
No	20%	61%	-0.920	20%	19%	0.034
Yes	80%	39%	0.920	80%	81%	-0.034
**Employment Status**						
Unemployed	8%	7%	0.019	8%	7%	0.040
Student	3%	4%	-0.076	3%	3%	0.003
Work at home/housewife	42%	44%	-0.028	42%	44%	-0.045
Farmer	25%	17%	0.190	25%	24%	0.032
Employed	23%	28%	-0.126	23%	23%	-0.005

Note: Estimates average across 100 imputed datasets.

* The number of matched CASG and non-CASG ranged from 6,609 to 6,662, with an average of 6,648.

### CASG and non-CASG Patient Outcomes

**Fig 2:**
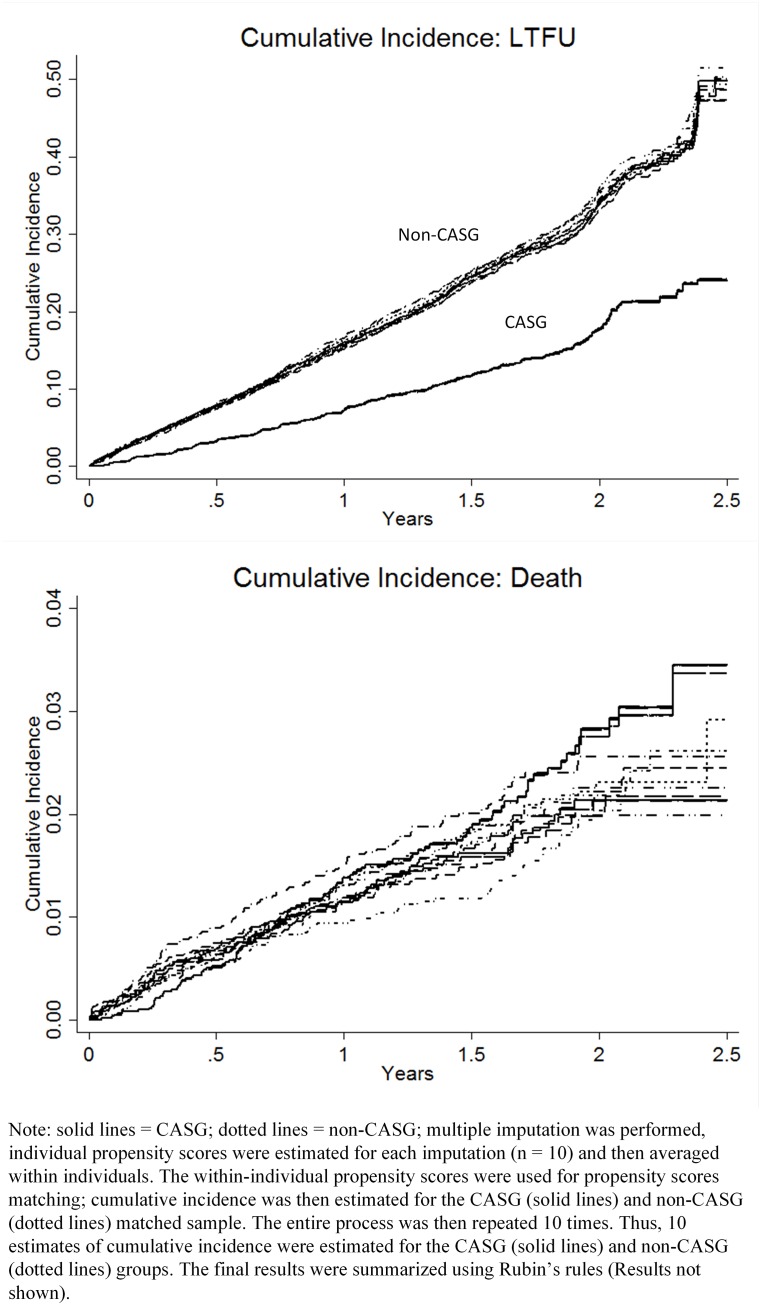
Cumulative incidence of LTFU and Death among matched CASG and non-CASG participants.

One year retention among CASG and non-CASG patients was 91.4% and 82.9%, respectively. Mortality among CASG and non-CASG patients was 1.4% and 1.2%, respectively. LTFU among CASG and non-CASG patients was 7.2% and 15.9%, respectively. Compared with eligible CASG participants, eligible non-CASG participants had significantly higher LTFU (hazard ratio [HR]: 2.36; 95% confidence interval [CI]: 1.54–3.17; *p* = .04; Fig 2). However, there were no significant differences in mortality between CASG and non-CASG participants (HR: 0.98; 95% confidence interval [CI]: 0.14–1.82; *p* = .96; reference group = CASG).

### Eligible versus Ineligible CASG participants

One year retention in eligible and ineligible CASG participants was 92.5% and 86.4%, respectively. Mortality in eligible and ineligible CASG participants was 0.8% and 4.0%, respectively. LTFU in eligible and ineligible CASG participants was 6.7% and 9.6%, respectively. Compared with eligible CASG participants, ineligible CASG participants had significantly higher mortality (HR: 4.519; 95% CI: 3.032–6.737; *p*<.001) and marginally higher LTFU (HR: 1.465; 95% CI: 0.956–2.243; *p* = .079).

## Discussion

During national roll-out, non-CASG participants had higher LTFU rates (p = 0.04) than matched CASG participants however there were no significant mortality differences between CASG and non-CASG participants. Thus, overall patients enrolled in CASG have significantly better retention than matched patients not enrolled in CASG. This evaluation demonstrates widespread implementation of CASG by the Ministry of Health is feasible and effective. This model need not remain within the confines of small, partner-managed initiatives to be successful, but rather can expand into the public space and be led, driven and implemented by governments. Other examples of successful community-based ART distribution have been reported in Uganda, Kenya, and Rwanda but most rely upon a distinct cadre of community health workers, who may or may not be patients.[21,22,23]

What is unique about the CASG is the inherent responsibility it confers upon patients for their own care and support, the relatively low financial investment required for the system to function, and the impact CASG expansion may have upon the absorptive capacity of the health system. Additional costs incurred by the health system were minimal and included trainings for providers on the CASG model, supervision of implementation of the CASG model at various levels of the health system. Qualitative evaluations focusing on cost savings incurred by both patients and the health system as well as perspectives and experiences from patients and providers when CASG is implemented are underway.

Since ART distribution in Mozambique occurs monthly, when six people on ART are grouped in a CASG, the number of routine yearly facility visits decreases from twelve to two. Given the financial and opportunity costs assumed by poor patients during a clinic visit, the impact of this reduction is significant.[24] CASG members who experience difficulty taking their medications are counseled by other members of the group. Improved adherence, increased motivation and confidence, and mutual support result in a higher level of physical, psychological and social well-being and improved health outcomes.[25]

CASG does not appear to confer a significant mortality benefit. A possible explanation is that the median time on ART for patients in this CASG cohort was 2.3 years prior to joining a CASG. The background mortality rate of patients on ART for >2 years is relatively low so it is not surprising that membership in a CASG does not confer demonstrable benefit.[26] In addition, it is likely that the accurate reporting of mortality amongst CASG members creates the impression of elevated mortality when in fact mortality rates amongst those LTFU is improperly counted as LTFU since the outcome is unknown.

### The Potential of Community-Led, Patient-Centered Interventions

The success of the CASG innovation in Mozambique could reshape the manner in which governments approach chronic care of HIV-infected patients. As high-burden countries tackle the pandemic, governments must respond to both the structural constraints inherent in weak public health systems and the day-to-day realities of poor, HIV-infected patients. Traditional facility-based models will be hard pressed to absorb the large numbers of HIV-infected patients in need of care, and health systems must begin to design models of chronic disease management that take both patient and system needs into account. Task shifting, decentralization, and patient empowerment are three essential elements of a successful model but none alone is sufficient to surmount the challenges faced by health systems that must provide lifelong care to large numbers of people in resource constrained settings. A combination of the three offers what we consider is the best way forward. Mozambique’s bold move to institutionalize a patient-centred approach to ART distribution is an example for other countries in the region to follow.

### Limitations

As a retrospective, observational cohort analysis, this evaluation is subject to residual confounding. By choosing to join a CASG, patients might be inherently different than eligible non-CASG patients in ways not accounted for using the propensity matching algorithm. There may be differential ascertainment of LTFU outcome between CASG and non-CASG patients. Undocumented transfers between facilities and unreported deaths among non-CASG persons are likely more frequently categorized as LTFU, whereas any transfer out or death of a CASG member would be reported as such and not categorized as LTFU. The data reviewed came from health facilities with Electronic Patient Tracking Systems (EPTS), which tend to be larger than those without EPTS. The programmatic criteria for patient participation in CASG excludes patients with poor adherence, requires that a patient be on ART for at least 6 months and have a CD4 count above 200 cells/m3. These criteria may exclude patients at highest risk for default and death.

## Conclusion

CASG participants are significantly less likely to be LTFU than matched non-CASG participants. Although we were not able to detect a mortality benefit, we suspect this is in part due to improved mortality reporting in the CASG population. These results support the Mozambican HIV program’s decision to scale this model up to the entire country and demonstrate that a successful CASG program can be implemented on a large-scale by the MoH with support from implementing partners. The CASG represents an innovative and effective new model for ART service provision in resource limited settings and we hope these results will prompt other national HIV programs to design and implement similar interventions. Evaluations of CASG in additional populations are urgently needed to determine whether the dynamic can help improve patient outcomes in other populations at risk for LTFU.
