# Correction: Community ART Support Groups in Mozambique: The Potential of Patients as Partners in Care

**DOI:** 10.1371/journal.pone.0176551

**Published:** 2017-04-20

**Authors:** Kebba Jobarteh, Ray W. Shiraishi, Inacio Malimane, Paula Samo Gudo, Tom Decroo, Andrew F. Auld, Vania Macome, Aleny Couto

The following information is missing from the Funding section: This research has been supported by the President’s Emergency Plan for AIDS Relief (PEPFAR) through the U.S. Centers for Disease Control and Prevention. The funder had no role in study design, data collection and analysis, decision to publish, or preparation of the manuscript.

The following information is missing from the Competing Interests section: This research was presented in part at the 20th International AIDS Conference, Melbourne, Australia, 20–25 July, 2014. The findings and conclusions in this report are those of the authors and do not necessarily represent the official position of the U.S. Centers for Disease Control and Prevention. This does not alter our adherence to all the PLOS ONE policies on sharing data and materials.
